# Toxicity of resin-matrix composites in a dental clinical setting

**DOI:** 10.1007/s10266-025-01055-y

**Published:** 2025-03-15

**Authors:** Maria Cordeiro, Júlio C. M. Souza, Ana T. P. C. Gomes, Patrícia Correia, Rita Fidalgo-Pereira

**Affiliations:** 1 Faculty of Dental Medicine, Universidade Católica Portuguesa, Estrada da Circunvalação, 3504-505 Viseu, Portugal; 2 Faculty of Dental Medicine, Center for Interdisciplinary Research in Health, CIIS, Universidade Católica Portuguesa, 3504-505 Viseu, Portugal; 3 Centro de Investigação em Tecnologias e Serviços de Saúde, UFP@RISE, Rede de Investigação em Saúde, Faculty of Health Sciences, Universidade Fernando Pessoa, Porto, Portugal; 4 PerMed Research Group, Center for Health Technology and Services Research (CINTESIS), Porto, Portugal; 5 Center for MicroElectroMechanical Systems (CMEMS-UMinho), University of Minho, 4800-058 Guimarães, Portugal; 6 LABBELS - Associate Laboratory, University of Minho, Guimarães, 4710-057 Braga, Portugal

**Keywords:** Toxicity, Resin monomers, BPA, Resin-matrix composite, Dentistry

## Abstract

The aim of this study was to perform a systematic review to comprehensively explore the factors contributing to the resin-matrix composites′ toxicity in patients. A systematic review was performed according to the PRISMA guidelines. The bibliographic review was performed in the following databases: PubMed, Cochrane Central and Web of Science. Inclusion and exclusion criteria were established to retrieve articles published in English over the last 20 years. The research yielded 1261 articles, with 1227 articles remaining after removing duplicates. Following a title and abstract screening, 20 articles underwent full-text review, and 12 in vivo studies were included. Detectable levels of free monomers and potential toxicity exposure were reported in the selected studies on saliva, urine, and blood samples. The selected in vivo studies suggest relatively low local and systemic toxicity although the available methods show technical limitations and therefore further studies are required. Dental operator-dependent factors were also identified. Standard chair-side guidelines on handling of resin-matrix composites placement plays a key role on the properties of the materials. The results reported noticeable changes in samples were not considered significant to affect patient's health, although, manufacturer’s instructions must be followed whilst using resin-matrix composites. In fact, adequate light curing parameters maintain a high degree of conversion of the resin-matrix composites  decreasing the release of residual monomers and thus the probability of related  toxicity.

## Introduction

Despite the adequate mechanical and optical properties, resin-matrix composites have shown some drawbacks such as: polymerization shrinkage, the release of monomers from the organic matrix due to incomplete monomers conversion during the polymerization, and material degradation over time [1, 2]. Resin-matrix composites are composed of an organic matrix, inorganic filler particles, a photoinitiator system, and silane coupling agents. Nano- and micro-scale inorganic filler particles are combined in resin-matrix composites to provide enhanced mechanical properties [3, 4]. However, concerns have arisen regarding potential toxicity related to materials applied in resin-matrix composites's organic matrix, which may include derivatives from bisphenol A (BPA). Namely, compounds such as bisphenol A-glycidyl methacrylate (Bis-GMA), along with, polycarbonate modified Bis-GMA (PC Bis-GMA), ethoxylated bisphenol A glycol dimethacrylate (Bis-EMA), and 2,2-bis[(4-methacryloxy polyethoxy) phenyl] propane (Bis-MPEPP) have raised concerns [5].

The effects of BPA are quite similar to those of two estrogens, diethylstilbestrol, and ethinyl estradiol, although with a lower potency level [6]. Due to their ability to simulate the actions of the estrogen hormone within living cells, BPA exhibits an affinity for binding to nuclear receptors, including estrogen, androgen, and thyroid hormone receptors. Additionally, BPA interacts with membrane receptors, potentially instigating endocrine-disrupting effects [7, 8]. A study conducted by Atkinson et al. [9] compared two commonly BPA derivatives in resin-matrix composites: Bis-DMA and Bis-GMA. In contrast to Bis-GMA, BPA has been identified in saliva due to the use of resin-matrix composites in the oral cavity. That occurs due to the hydrolysis of bisphenol catalyzed by salivary esterases, a result of inherent structural differences between those compounds. It is noteworthy that Bis-DMA is not widely applied in dental materials [8, 10]. Moreover, several studies have highlighted the cytotoxic effects associated with both TEGDMA and UDMA [9]. The release of monomers and BPA is dependent upon the chemical composition and content of the organic matrix present in resin-matrix composites [3]. TEGDMA is a genotoxic monomer known to impact DNA even at low concentrations, causing fragmentation and destruction by apoptosis induction leading to activation of caspases -3, -8, and -9. Lipopolysaccharide-induced apoptosis decreases the release of IL-1β and TNF-α, with more pronounced effects at higher concentrations. The low molecular weight of the monomer has been associated with reduced glutathione levels, providing protection against reactive oxygen radicals [8, 11]. Research has shown the presence of HEMA and TEGDMA monomers at microgams in saliva within minutes to hours, after the placement of a resin-matrix composite. Furthermore, those monomers have been detected in the dentine and pulp after hours to days of the resin-matrix composite placement [12]. Long-term exposure to HEMA, even at low concentrations, may result in immune suppression and clastogenic effects. Higher concentrations of residual monomers have been associated with significant reductions in IgG1 and IgM production and adverse effects on cell proliferation [11, 13]. The cytotoxicity assessment of such monomers was conducted using the bromodeoxyuridine and the lactate dehydrogenase assays. A previous study showed the highest degree of toxicity for Bis-GMA followed by UDMA and TEGDMA, while HEMA revealed the lowest degree of toxicity [14].

**Fig. 1:**
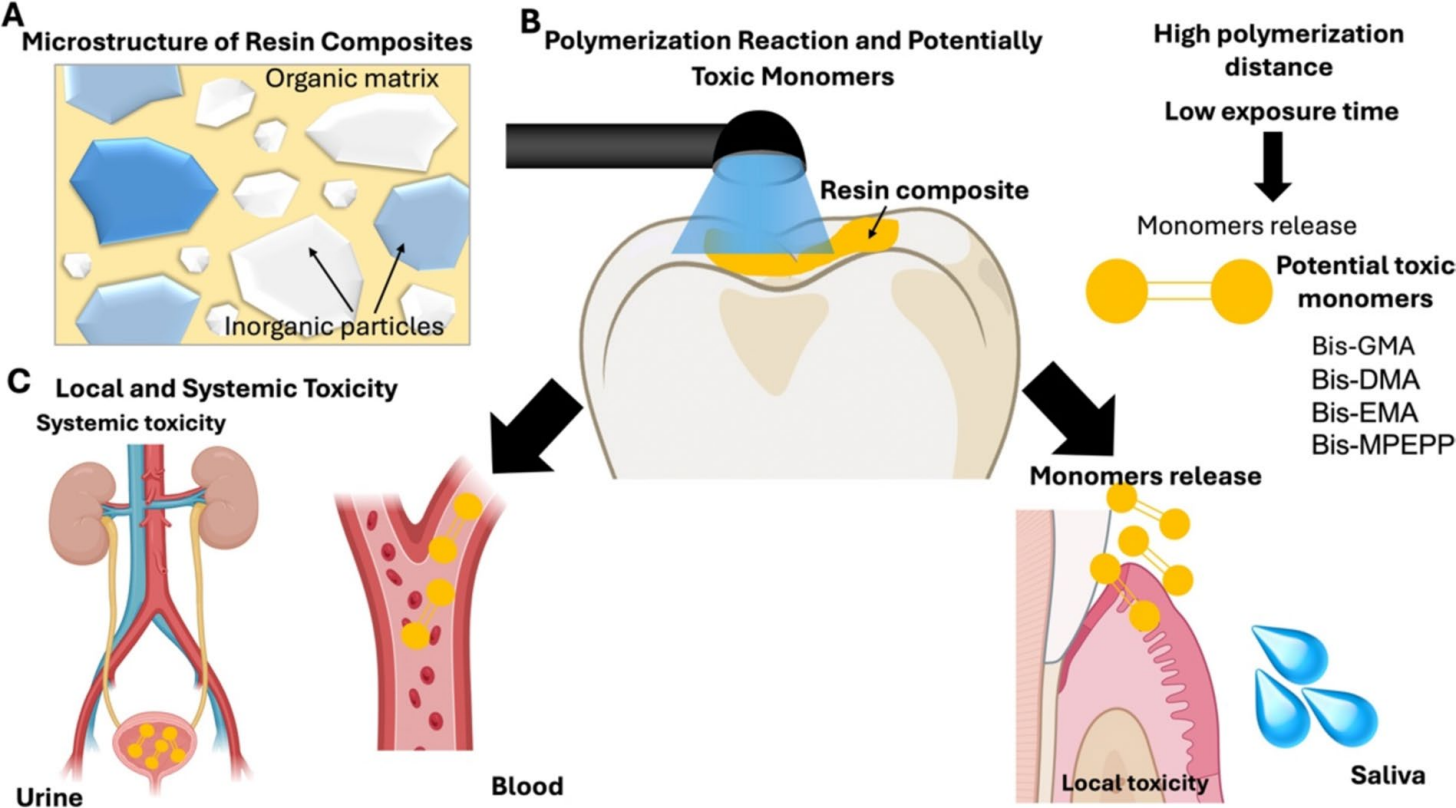
Schematics of monomers release and potential toxicity. **A** resin-matrix composites microstructure, **B** Polymerization reaction and potential toxic monomers, **C** Local and systemic toxicity

Regarding the factors that influence toxicity, the light curing procedures, type and size of fillers and organic matrix composition may also have an impact on the resin composites toxicity [15] Fig. 1.

Also, toxicity of resin composites may be caused by degradation of the organic matrix due to wear caused by occlusal forces during mastication. The impact of saliva constituents via hydrolytic and enzymatic pathways is also an important factor. The resin-matrix composites toxicity is intrinsically related to synergistic factors within the oral cavity, temperature fluctuations, and the acidic by-products synthesized by oral microbial activity [16, 17].

The potential toxicity of specific components in resin-matrix composites demands ongoing, comprehensive evaluation of their physicochemical properties and biological reliability for complete clinical acceptance. Prior research has highlighted the toxicity risks associated with resin-matrix composites at both local and systemic levels, underscoring the critical importance of continued investigation in this area [1, 18].

The aim of the present study was to perform a systematic review of the local and systemic toxicity of resin-matrix composites in patients.

## Method

### Search strategy

This systematic review was registered on PROSPERO (International Prospective Register of Systematic Reviews) platform, with the number 480689, following the standard guidelines outlined by PRISMA (Preferred Reporting Items for Systematic Reviews and Meta-Analyses). The present systematic review was carried out in accordance with previous systematic reviews [19, 20]. A research question has been formulated following the PICO strategy, designated by P (population), I (intervention), C (comparison) and O (outcome): Which factors influence the toxicity of resin-matrix composites for dental restorations, and what are their local or systemic adverse effects in humans? The literature search was conducted across three online scientific platforms, including PubMed, Web of Science and Cochrane Central (Table 1, 2 and 3). The search filters specifically targeted articles in English, published in the last 20 years and studies conducted in humans. In the PubMed search, both free vocabulary and MeSH terms were used to refine and enhance the precision of the search, as seen in Table 1. The inclusion criteria were in vivo studies/Humans, Randomized Controlled Trial (RCT), and Toxicity Studies. The exclusion criteria were in vitro and animal studies, other composite materials, systematic reviews, metanalyses, narrative reviews, clinical case reports and case series. The following equations applied on the three databases are shown in Table 1, 2 and 3:

**Table 1:** Keywords applied on Pubmed database

Pubmed	
#1	(toxicity) OR (toxic)) OR (cytotoxicity)) OR (toxicology)) OR (BPA)) OR (Bis-GMA)) OR (Bisphenol-A)) OR (Bis-DMA)) OR (UDMA)) OR (TEGDMA)) OR (polymers)) OR (comonomers)) OR (copolymers)) OR (resin monomers)) OR (resin conversion)) OR (component release)) OR (bpa-derivatives)) OR (bisphenol a-glycidyl methacrylate)) OR (2,2-di(4-methacryloxyphenyl) propane)) OR (2,2-bis-(4-(2-methacryloxyethoxy)phenyl)propane)) OR (toxicology[MeSH Terms])) OR (Bisphenol A-Glycidyl Methacrylate[MeSH Terms])) OR (2,2-di(4-methacryloxyphenyl) propane[MeSH Terms])) OR (2,2-bis-(4-(2-methacryloxyethoxy)phenyl)propane[MeSH Terms])) OR (polymers[MeSH Terms])
#2	(((((((((((resin-matrix composite) OR (resin composite)) OR (resin-based composites)) OR (dental composite resin)) OR (composite resins)) OR (resin-matrix composition)) OR (different composition resins)) OR (flow resin)) OR (bulk-fill resin composites)) OR (resin nanocomposites)) OR (conventional composite resin)) OR (composite resins[MeSH Terms])
#3	(((((Dentistry) OR (oral)) OR (mouth)) OR (dental)) OR (dentistry[MeSH Terms])) OR (mouth[MeSH Terms])
#4	((((((((((health) OR (oral health)) OR (human's health)) OR (biosafety)) OR (immunological effect)) OR (immune system)) OR (allergy)) OR (hypersensitivity)) OR (toxicity)) OR (cytotoxicity)) AND (release)
	#1 AND #2 AND #3 AND #4

**Table 2:** Keywords applied on Web of Science database

Web of Science	
#1	ALL = (toxicity OR toxic OR cytotoxicity OR toxicology OR BPA OR Bis-GMA OR Bisphenol A OR Bis-DMA OR UDMA OR TEGDMA OR polymers OR comonomers OR copolymers OR resin monomers OR Resin conversion OR component release OR bpa-derivatives OR bisphenol a-glycidyl methacrylate OR 2,2-di(4-methacryloxyphenyl) propane OR 2,2-bis-(4-(2-methacryloxyethoxy)phenyl)propane)
#2	ALL = (resin-matrix composite OR resin composite OR resin-based composites OR dental composite resin OR composite resins OR resin-matrix composition OR different composition resins OR flow resin OR bulk-fill resin composites OR resin nanocomposites OR conventional composite resin)
#3	ALL = (Dentistry OR Dental OR oral OR mouth)
#4	ALL = ( in vitro OR animal study OR cell culture OR tissue culture OR animal model OR laboratory animals OR cell cultivation OR In Vitro Techniques OR Animal Experimentation OR Cell Culture Techniques OR Tissue Culture Techniques
#5	ALL = (adhesive OR cement OR fillings OR orthodontic adhesive OR sealants OR adhesives OR Cements, dental OR Dental Sealants OR Pit and Fissure Sealants OR Root Canal Filling Materials
	#1 AND #2 AND #3 NOT #4 NOT #5

**Table 3:** Keywords applied on Cochrane Central database

Cochrane Central	
#1	toxicity OR toxic OR cytotoxicity OR toxicology OR BPA OR Bis-GMA OR Bisphenol A OR Bis-DMA OR UDMA OR TEGDMA OR polymers OR comonomers OR copolymers OR "resin monomers" OR "resin conversion" OR "component release" OR bpa-derivatives OR "bisphenol a-glycidyl methacrylate"
#2	"resin-matrix composite" OR "resin composite" OR "resin-based composites" OR "dental composite resin" OR "composite resins" OR "resin-matrix composition" OR "different composition resins" OR "flow resin" OR "bulk-fill resin composites" OR "resin nanocomposites" OR "conventional composite resin"
#3	dentistry OR Dental OR oral OR mouth
#4	"in vitro" OR "animal study" OR "cell culture" OR "tissue culture" OR "animal model" OR "laboratory animals" OR "cell cultivation" OR "In Vitro Techniques" OR "Animal Experimentation" OR "Cell Culture Techniques" OR "Tissue Culture Techniques"
#5	adhesive OR cement OR fillings OR "orthodontic adhesive" OR sealants OR Adhesives OR "Cements, dental"OR "Dental Sealants" OR "Pit and Fissure Sealants" OR "Root Canal Filling Materials"
	#1 AND #2 AND #3 NOT #4 NOT #5

### Study selection and data collection process

The publications obtained from the three databases (PubMed, Cochrane Central, and Web of Science) were exported to the “Rayyan’s Intelligent Systematic Review Platform”[21], of which duplicates were excluded. The studies' details  and numerical data were extracted using a customized Excel file. The following variables were collected for this review: authors' names, publication year, country, sample size/age range, aims, resin-based materials, methodology, monomers source materials, local or systemic toxicity and main outcomes (Table S1, suplementary material). Cohen's kappa coefficient was used to assess the risk of bias among researchers, through the selection process. The Revised Cochrane risk-of-bias tool for randomized trials (RoB 2)[22] was also used to assess the quality of the studies. The Cochrane Risk of Bias tool (RoB2) was used to assess the risk of bias in the studies, Fig. 3. Except for three studies that demonstrated “some concerns”[23-25], the remaining 9 studies [26-33] demonstrated “low risk of bias”.

## Results

**Fig. 2:**
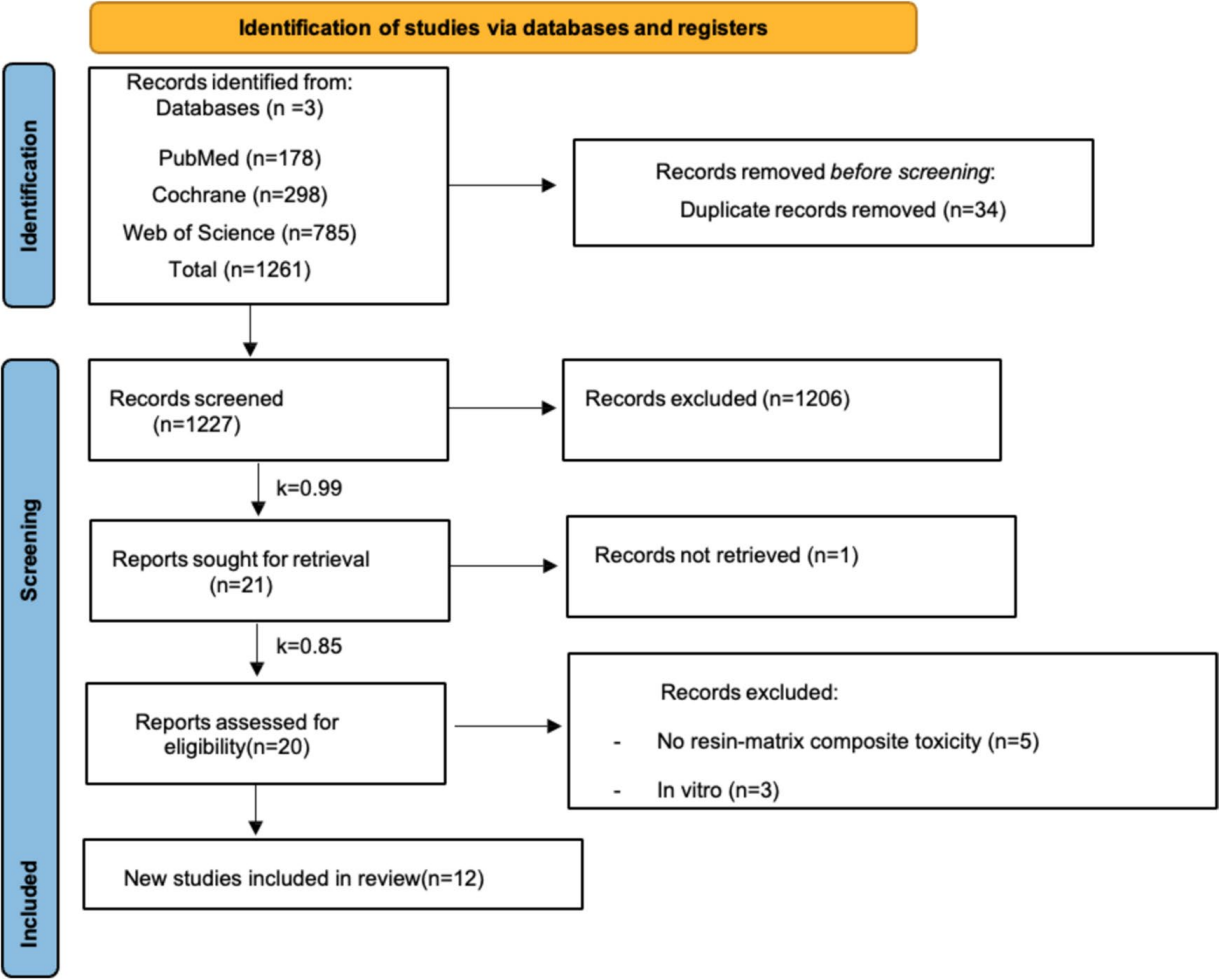
PRISMA flow diagram with research data. k – Cohen´s k

A total of 1261 articles were identified on three electronic databases. Following the removal of duplicates, a total of 1227 articles remained for further selection by title and abstract. Twenty one articles records were sought for retrieval although 1 article was not provided. Then, 20 articles were subjected to full reading and 12 articles were included while 8 studies were excluded. The results of the selection of articles are shown in Fig. 2.

Cohen's kappa coefficient was used to assess the agreement among researchers, giving in the first part of the selection (title and abstract) Cohen's k = 0.99. Almost a complete agreement (99.8%) and in the second part of the selection (full reading) Cohen's k = 0.85 (Fig. 2).

In the present systematic review, all included articles investigated the toxicity of specific monomers attributed to resin composites. To determine the quantity of detected monomers and their toxicity, analyses of saliva, urine, and blood were performed. Regarding the analytical methods, two articles (16.66%) focused solely on urine analysis [26, 27], three studies (25%) exclusively on saliva analysis, and two studies (16.66%) on blood analysis [26, 34, 23, 24,25,28,29]. One article (8.33%) examined both urine and saliva samples, while another study (8.33%) evaluated both urine and blood [30, 31]. Various materials were used for analysis, such as resin-matrix composites, occlusal pit and fissure sealants, compomers, and adhesives. The remaining three articles (23%) utilized distinct toxicity assessment methods. One study applied skin-patch testing, while another one used neuropsychological tests, and a third one applied tests to evaluate the physical development of children [27, 32, 35].

The  detailed retrieved data are listed on S1 (supplementary material), and the main results were evaluated and described below:

- In five studies, Z100^™^ (3 M ESPE) resin-matrix composites were assessed, which contained Bis-GMA and TEGDMA monomers, except for one that only contained Bis-GMA [25, 28, 31, 32,35].
- The Z250 ^™^ (3M ESPE) resin-matrix composite  was evaluated in one study, which specified the Bis-GMA, Bis-EMA, TEGDMA and UDMA monomers [29].
- One study assessed Tetric Evoceram^™^  (Ivoclar-Vivadent) resin-matrix composite which contains Bis-GMA, UDMA, and TEGDMA [30].
- In one of the studies, Charisma™ (Heraeus Kulzer) resin-matrix composite was studied, and the microstructure contained combined monomers such as, Bis-GMA and TEGDMA. Additionally, Grandio^™^ resin-matrix composite was investigated and included Bis-GMA, TEGDMA, and UDMA [24].
- One of the studies assessed six different resin-matrix composites brands that were not used in the other studies: Progress^™^ (Kanebo) and Metafil Flo (Sun Medical) with UDMA and TEGDMA; Palfique Toughwell^™^ (Tokuyama) and Xeno CFII^™^ (Sankin Kogyo) only with Bis-GMA; Beautifil™ (Shofu), Prodigy^™^ (Kerr), and Clearfil ST (Kuraray) with Bis-GMA and TEGDMA in their chemical composition [25].
- In two studies, a Revolution^™^ (Kerr) flowable resin-matrix composite was investigated, containing solely the Bis-GMA monomer in its chemical composition [28, 31].
- In three studies, a Dyract™ (Dentsply) compomer was assessed. The indicated chemical composition was different between studies. In one, the chemical composition was reported as UDMA and TEGDMA, while another study reported only UDMA. In the third study, the compomer was composed of UDMA and trimethacrylate resins [28, 32, 35]. A sealant of occlusal pits and fissures was analyzed in two studies, using Ultraseal XT^™^  (Ultradent). In both studies, Bis-GMA was present in their chemical composition, but only one contained UDMA [28, 31].
- In addition to the aforementioned materials, adhesives were also incorporated into two studies, each study utilized a different adhesive. One applied Clearfil S3 Bond^™^ (Kuraray), containing Bis-GMA and TEGDMA monomers. The other study assessed Unifil^™^(GC), containing solely the UDMA monomer [24, 25].
- In the skin patch testing study, the following monomers were analyzed MMA, TEGDMA, EGDMA, Bis-GMA, 2-HEMA (0.2% pet, Chemotechnique Diagnosis) and formaldehyde [27].
- However, in three studies there were no specifications regarding the brands or types of materials used. Among those, two studies solely reported the BPA evaluation without specifying the chemical composition of the resin-matrix composites. The third study explicitly stated the analysis of Bis-GMA but did not mention the material [26, 34, 23].
- Two of the selected articles investigated the influence of light-curing on local or systemic toxicity [24, 30]. Another study mentioned a light-curing duration of 60 s without specifying the light-curing unit or intensity used [25].

**Fig. 3:**
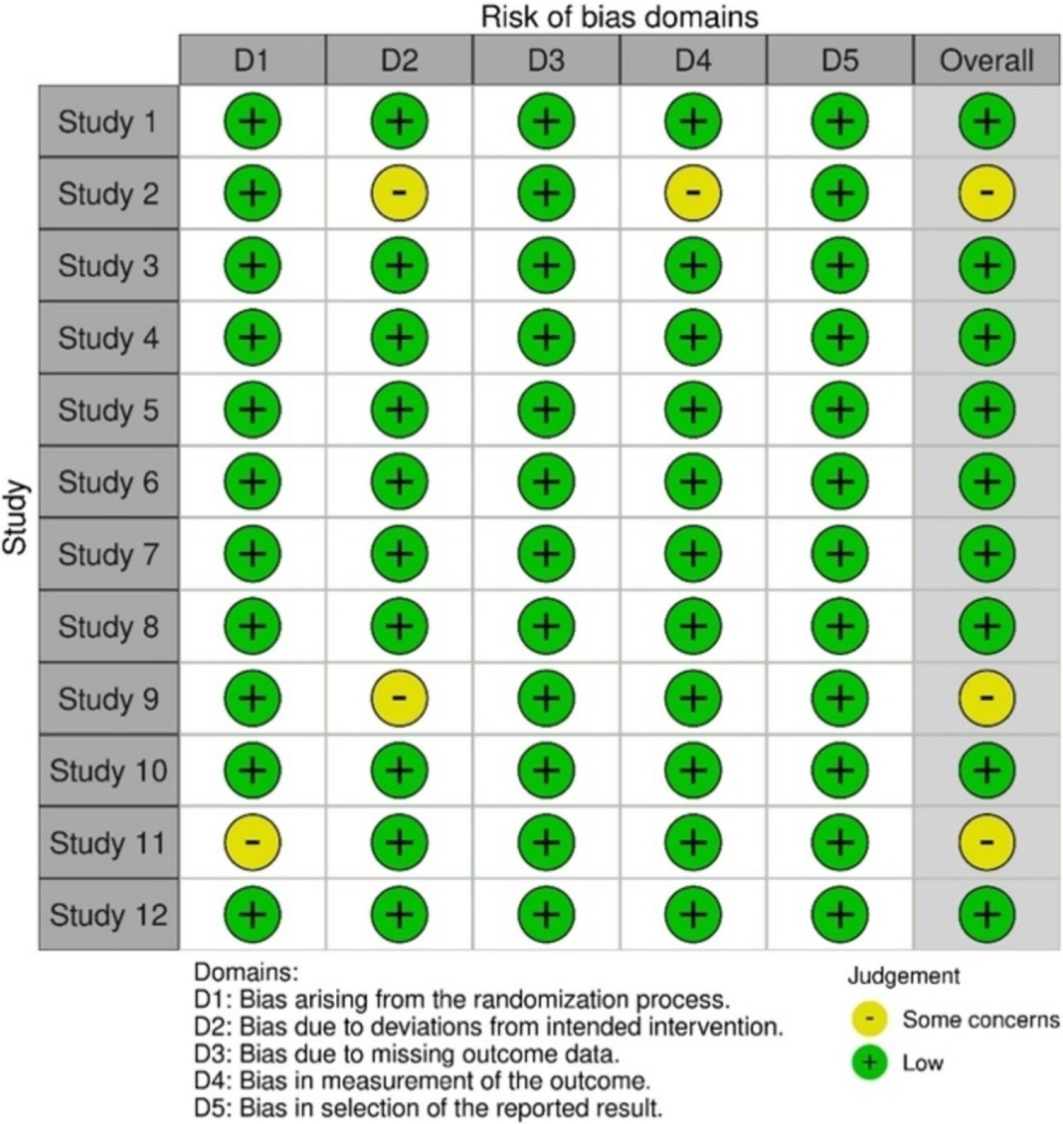
Risk of bias assessment with RoB 2

The Cochrane Risk of Bias tool (RoB2) was used to assess the risk of bias in the studies (Fig. 3). Except for three studies that revealed  some concerns [23–25], the remaining 9 studies [26–33] revealed low risk of bias.

## Discussion

The present systematic review reported the major findings from previous studies regarding the toxicity of resin-matrix composites. This study gathered relevant information on local and systemic toxicity related to the chemical composition of resin-matrix composites. Additionally, it included the relevant factors associated with resin-matrix composites' toxicity, such as short exposure time on light-curing and adequate precautionary measures to decrease the resin-matrix composites potential toxicity.

### Relationship between the chemical composition of resin-matrix composites and potential toxicity

The content of monomers released from resin-matrix composites is influenced by the polarity of the solvent, the polymerization degree of the material, and variations in the chemical composition and filler-monomer ratio. All of those factors significantly impact the release of monomers and the cytotoxicity of the restorative material [36, 37].

In the study conducted by Sideridou et al. [38], UDMA and Bis-GMA exhibited significantly higher initial polymerization reactivity compared to TEGDMA and Bis-EMA. Within 10 s of polymerization, UDMA and Bis-GMA achieved reaction percentages of 49.5% and 22.9% of their double bonds, respectively, while TEGDMA and Bis-EMA only reached 13.7% and 13.5%, respectively. However, as polymerization progressed, TEGDMA and Bis-EMA displayed a markedly higher rate of polymerization than UDMA and Bis-GMA. Ultimately, TEGDMA exhibited the highest conversion rate. This phenomenon may be due to differences in the chain structure of the linking group between the methacrylate groups. It is noteworthy the distinct chemical properties and reactivity potential between Bis-GMA and TEGDMA, with Bis-GMA being considered more reactive due to its steric structure, which hinders a higher degree of conversion (DC).  Such findings align with the study of Geurtsen et al. [39] since TEGDMA exhibits the highest DC, and therefore it is considered to be less prone to toxicity. Conversely, Bis-GMA is characterized by a lower DC, due to a higher monomer viscosity and it is associated with an increased level of toxicity [29, 38, 40, 41]. It has been established that the predominant monomer is HEMA, due to its size and minimal molecular weight. The high molecular weight of Bis-GMA, coupled with its substantial dimensions and limited solubility in water leads to the release of low concentrations. Nevertheless, it has been noticed that a high monomer release occurs in an organic solvent environment compared to a water-based solution [42]. Toxicity is also influenced by molecular weight since higher molecular weights correspond to higher  toxicity. The molecular weights of prevalent monomers such as HEMA (130 g mol^−1^), TEGDMA (286 g mol^−1^), UDMA (471 g mol^−1^), and Bis-GMA (512 g mol^−1^), can indicate the toxic potential of the monomer [29, 39].

Maserejian et al. [28] studied the changes in immune function related to resin-matrix composites and analyzed a flowable resin-matrix composite. As demonstrated in the investigation by Wataha et al. [43], several flowable resin-matrix composites were characterized by reduced filler content and elevated monomer concentration in comparison to ordinary materials. The high toxicity potential may be attributed to the increased organic matrix content of flowable resin-matrix composites, and lower inorganic content comparatively to ordinary resin-matrix composites. Also, flowable resin-matrix composites reveal lower mechanical properties leading to increased degradation. However, conventional resin composites applied in high-thickness layers can achieve an incomplete polymerization, leading to increased release of residual monomers [43, 44].

Bakopoulou et al. [33] analyzed the impact on the induction of sister chromatid exchange (SCE), chromosome aberrations (CAs), as well as effects on cell cycle kinetics and mitotic indices of human peripheral lymphocytes. Notably, Tetric Ceram^TM^ exhibited the highest SCE induction and Filtek Z250^™^ was associated with cell cycle delays and a reduction in the mitotic index [33].

### Precautionary measures to avoid toxicity caused by resin-matrix composites

Although the selected studies reported that BPA toxicity did not reach high levels, precautionary measures must be followed when performing a dental restoration, such as adequate polymerization exposure time, short polymerization distance to minimize incomplete polymer chain formation.

Several factors have been identified that can influence the DC of monomers and thus enhance toxicity. Such factors include the optical properties of the resin-matrix composite, the duration/intensity/distance of light curing, the thickness of the material increment, the light curing method used, and the type of inorganic filler. Adequate polymerization is considered the primary criterion for a successful restorative procedure [45].

Regarding the optical properties of the resin-matrix composite, several studies have shown that translucency may have an effect on polymerization time. Specifically, a longer polymerization time is recommended for darker shades. More translucent resin-matrix composites provide  high light transmission and, consequently, higher DC of monomers. Therefore, for darker shades, a longer polymerization time is advisable to achieve adequate polymerization energy and enhance the DC of monomers [46, 47].  On light curing time, it has been demonstrated that extending the light exposure beyond the manufacturer's recomendations by an additional 10–20 s has positive effects on the DC  of monomers [48]. On bulk-fill resin-matrix composites, increments of 2 to 4 mm can be placed while maintaining the appropriate DC. That occurs due to the greater translucency and consequently light transmission compared to ordinary resin-matrix composites [49, 50]. Bulk-fill resin-matrix composites exhibit larger inorganic filler sizes, leading to a reduced inorganic filler-matrix interface, thereby enhancing the transmission of polymerization light and resulting in a higher DC. Additionally, such resin-matrix composites have lower inorganic filler content, which contribute to increased translucency and, consequently, higher DC of monomers compared to flowable and ordinary resin-matrix composites [51, 52].

However, some measures can be applied to prevent resin-matrix composites' toxicity. One study found that applying rubber dams reduces BPA levels in saliva. Results indicated that concentrations of BPAHPE and Bis-GMA within one hour post-treatment were two to four times higher in participants without a rubber dam compared to those with one. However, BPA concentrations in saliva returned to pre-treatment levels for 1 and 8h post-treatment [53]. In another investigation, the application of pumice on a cotton roll or by a prophylactic rubber cup was found to be notably effective in mitigating the absorption of Bis-DMA, Bis-GMA, and TEGDMA compared to dry or moist cotton rubbing, or the use of an air/water spray [10]. Regarding light-curing exposure time, a study concluded that on a polymerization time less than 40 s, and a temperature of about 37 ◦C in the oral cavity, resin-matrix composites are never polymerized to their full extent, as the propagation of the cross-linking reaction drastically reduces the mobility of monomers [38]. Furthermore, other studies have  recomended rinsing for 30 s post-polymerization substantially diminishes the amount of BPA released [10, 25]. Enhanced risk management strategies and effective occupational health and safety programs, are essential for oral health students and professionals daily exposed to methacrylates [27].

The study of Maserejian et al. [28] examined alterations in children's immune responses following resin-matrix composite restoration. The results showed changes in B-cell reactivity, while monocyte reactivity exhibited a decline within the initial six-month period. Moreover, those alterations were particularly pronounced in children with a higher prevalence of Bis-GMA-based resin-matrix composite restorations [28]. The study by Yıldız et al. [29] investigated the correlation between resin-matrix composites and the release of substances into the oral cavity, assessing their impact on lipid peroxidation and DNA oxidation through blood circulation monitoring. Results revealed a significant elevation in the 8-OHdG/106 dG ratio within the resin-matrix composite filling cohort. Furthermore, Bis-GMA and TEGDMA increased markers of lipid peroxidation and DNA oxidation [29]. The selected articles focused on local and systemic toxicity following distinct methods. Regarding sample size and age range, the studies analyzed different age ranges and distinct sample sizes. Berge et al. [30] analyzed 20 patients with ages between 16 and 40 years, while Chung et al. [26] analyzed 495 children with ages between 8 and 9 years, Yıldız et al. [29] studied 41 participants with ages between 17 and 23 years, and Maserejian et al. [32] that analyzed 5116 children with ages between 6 and 10 years. Regarding samples was difficult to establish a relationship between toxicity and patients’ age. Regarding materials, some studies did not provide the materials' chemical composition [26, 23]. The most frequent monomers found on the studied resin-matrix composites were: Bis-GMA, TEGDMA, and UDMA, followed by HEMA and Bis-EMA in lower content. It should be highlighted  the lack of methodology regarding the restorative procedures, especially the light-curing unit, exposure time, polymerization distance, and the type of isolation used, i.e. rubber dam. Those factors are essential to establish clinical guidelines to reduce the resin-matrix composite potential toxicity. Only one study [25] mentioned the type of polishing applied to resin-matrix composite restoration. Across the selected studies, concentrations of both BPA and monomers did not reach levels sufficiently high to elicit either local or systemic toxicity. Consequently, exposure to BPA within resin-matrix composite restorations remains low, limiting the potential risk of adverse effects. Furthermore, no significant association was established between BPA concentration levels and the area of resin-matrix composite restoration surfaces, nor were substantial alterations noted in the variables evaluated across the studies [23-26, 30–35]. In addition, long-term studies in humans are crucial to evaluate the toxicity levels over time to establish a possible relation with chronic effects. The present systematic review searched for evidence of toxicity in humans caused by resin-matrix composite monomers that distinguishes it from other reviews [7, 54]. Alterations related to resin-matrix composites´ toxicity can be evaluated within safety values not considered significantly relevant to substantially affect a patient's health.

Several limitations of the study can be identified. At first, the analysis was limited to specific monomers, excluding others that may also be potentially toxic. More high-quality studies, particularly randomized controlled clinical trials (RCTs), are required. Additionally, the heterogeneity of resin-matrix composite materials poses a challenge. There are confounding factors in measuring BPA levels, considering its presence in food and the environment. Furthermore, long-term assessments are essential to accurately confirm the analyzed results.

## Conclusion

This systematic review highlighted that traces of residual monomers are frequently detected in biological fluids of patients with resin-matrix composite restorations, underscoring the importance of applying precautionary measures.

The release of residual monomers is variable and depends on several patient- and material-related factos that should be carefully examined in further in vivo and in vitro studies. Precautionary measures must be followed before placing resin-matrix composites to guarantee an adequate polymerization reaching optimum properties of the materials. That results in a lower release of residual monomers, reducing the probability of toxicity.

Several factors add complexity to the subject with multiple sources of BPA via dietary and environmental exposures. Further clinical research is imperative to establish causality, particularly considering exposure to non-dental sources containing BPA.

## Data Availability

Data will be available on request.

## References

[1] Worthington H, Khangura S, Seal K, Mierzwinski-Urban M, Keenan A, Sahrmann P, et al. Direct composite resin fillings versus amalgam fillings for permanent posterior teeth. Cochrane Database Syst Rev. 2021. 10.1002/14651858.CD005620.pub3. 34387873 10.1002/14651858.CD005620.pub3 PMC8407050

[2] Peutzfeldt A. Resin composites in dentistry: the monomer systems. Eur J Oral Sci. 1997;105:97–116. 10.1111/j.1600-0722.1997.tb00188.x. 9151062 10.1111/j.1600-0722.1997.tb00188.x

[3] Bezgin T, Cimen C, Ozalp N. Evaluation of residual monomers eluted from pediatric dental restorative materials. Biomed Res Int. 2021;2021:6316171. 10.1155/2021/6316171. 34580639 10.1155/2021/6316171 PMC8464417

[4] Ferracane J, Pfeifer C, Hilton T. Microstructural features of current resin composite materials. Curr Oral Health Rep. 2014. 10.1007/s40496-014-0029-4.

[5] Dursun E, Fron Chabouis H, Attal J-P, Raskin A. Bisphenol a release: survey of the composition of dental composite resins. Open Dent J. 2016;10:446–53. 10.2174/1874210601610010446. 27708726 10.2174/1874210601610010446 PMC5039892

[6] Myers DE. Terms and conditions privacy policy Current status of potential bisphenol toxicity in dentistry. Gen Dent. 2011;59(4):262–5. 21903565

[7] Lopes-Rocha L, Ribeiro-Gonçalves L, Henriques B, Özcan M, Tiritan ME, Souza JCM. An integrative review on the toxicity of Bisphenol A (BPA) released from resin composites used in dentistry. J Biomed Mater Res B Appl Biomater. 2021;109:1942–52. 10.1002/jbm.b.34843. 33834604 10.1002/jbm.b.34843

[8] Löfroth M, Ghasemimehr M, Falk A, Vult von Steyern P. Bisphenol A in dental materials – existence, leakage and biological effects. Heliyon. 2019;5: e01711. 10.1016/j.heliyon.2019.e01711. 31193754 10.1016/j.heliyon.2019.e01711 PMC6538958

[9] Aydın N, Karaoglanoglu S, Oktay E, Kılıç SA. Evaluating cytotoxic effects of highly esthetic dental composites. Braz Dent Sci. 2020. 10.14295/bds.2020.v23i1.1883.

[10] Fleisch AF, Sheffield PE, Chinn C, Edelstein BL, Landrigan PJ. Bisphenol A and related compounds in dental materials. Pediatrics. 2010;126:760–8. 10.1542/peds.2009-2693. 20819896 10.1542/peds.2009-2693 PMC4139922

[11] Pagano S, Coniglio M, Valenti C, Negri P, Lombardo G, Costanzi E, et al. Biological effects of resin monomers on oral cell populations: descriptive analysis of literature. Eur J Paediatr Dent. 2019;20:224–32. 10.23804/ejpd.2019.20.03.11. 31489823 10.23804/ejpd.2019.20.03.11

[12] Hume WR, Gerzina TM. Bioavailability of components of resin-based materials which are applied to teeth. Crit Rev Oral Biol Med. 1996;7(2):172–9. 8875031 10.1177/10454411960070020501

[13] González-López JA, Pérez-Mondragón AA, Cuevas-Suárez CE, Trejo-Carbajal N, Herrera-González AM. Evaluation of dental composites resins formulated with non-toxic monomers derived from catechol. J Mech Behav Biomed Mater. 2020;104: 103613. 10.1016/j.jmbbm.2019.103613. 31929099 10.1016/j.jmbbm.2019.103613

[14] Walther U, Walther S, Liebl B, Reichl F, Kehe K, Nilius M, et al. Cytotoxicity of ingredients of various dental materials and related compounds in L2- and A549 cells. J Biomed Mater Res. 2002;63:643–9. 10.1002/jbm.10384. 12209911 10.1002/jbm.10384

[15] Fronza BM, Ayres A, Pacheco RR, Rueggeberg FA, Dias C, Giannini M. Characterization of inorganic filler content, mechanical properties, and light transmission of bulk-fill resin composites. Oper Dent. 2017;42:445–55. 10.2341/16-024-L. 28402731 10.2341/16-024-L

[16] Gupta S, Saxena P, Pant A, Pant A. Release and toxicity of dental resin composite. Toxicol Int. 2012;19:225–34. 10.4103/0971-6580.103652. 23293458 10.4103/0971-6580.103652 PMC3532765

[17] Bakopoulou A, Papadopoulos T, Garefis P. Molecular toxicology of substances released from resin-based dental restorative materials. Int J Mol Sci. 2009;10:3861–99. 10.3390/ijms10093861. 19865523 10.3390/ijms10093861 PMC2769064

[18] Pingale P, Saudagar N, Rajput A, Rajpoot K, Pingale A, Tekade R. Toxicity of dental materials and ways to screen their biosafety. In: Essentials of Pharmatoxicology in Drug Research, Volume 1. Elsevier; 2023. p. 435–68.

[19] Lopes PC, Carvalho T, Gomes ATPC, Veiga N, Blanco L, Correia MJ, et al. White spot lesions: diagnosis and treatment – a systematic review. BMC Oral Health. 2024;24:58. 10.1186/s12903-023-03720-6. 38195439 10.1186/s12903-023-03720-6 PMC10775501

[20] Lopes PC, Gomes ATPC, Mendes K, Blanco L, Correia MJ. Unlocking the potential of probiotic administration in caries management: a systematic review. BMC Oral Health. 2024;24:216. 10.1186/s12903-024-03893-8. 38341538 10.1186/s12903-024-03893-8 PMC10859023

[21] Ouzzani M, Hammady H, Fedorowicz Z, Elmagarmid A. Rayyan—a web and mobile app for systematic reviews. Syst Rev. 2016. 10.1186/s13643-016-0384-4. 27919275 10.1186/s13643-016-0384-4 PMC5139140

[22] Sterne JAC, Savović J, Page MJ, Elbers RG, Blencowe NS, Boutron I, et al. RoB 2: a revised tool for assessing risk of bias in randomised trials. BMJ. 2019;366: l4898. 10.1136/bmj.l4898. 31462531 10.1136/bmj.l4898

[23] Berge TLL, Lygre GB, Jönsson BAG, Lindh CH, Björkman L. Bisphenol A concentration in human saliva related to dental polymer-based fillings. Clin Oral Investig. 2017;21:2561–8. 10.1007/s00784-017-2055-9. 28181074 10.1007/s00784-017-2055-9

[24] Gul P, Celik N, Ozgeris FB, Demirkaya-Miloglu F, Kiziltunc A, Seven N. Effects of bisphenol A released from composite fillings on reproductive hormone levels in men. Int Dent J. 2021;71:343–51. 10.1016/j.identj.2020.12.008. 33583564 10.1016/j.identj.2020.12.008 PMC9275326

[25] Sasaki N, Okuda K, Kato T, Kakishima H, Okuma H, Abe K, et al. Salivary bisphenol-A levels detected by ELISA after restoration with composite resin. n.d. 10.1007/s10856-005-0627-8 15803273

[26] Chung SY, Kwon H, Choi YH, Karmaus W, Merchant AT, Song KB, et al. Dental composite fillings and bisphenol A among children: a survey in South Korea. Int Dent J. 2012;62:65–9. 10.1111/j.1875-595X.2011.00089.x. 22420473 10.1111/j.1875-595X.2011.00089.x PMC9374909

[27] Lyapina M, Dencheva M, Krasteva A, Tzekova M, Kisselova-Yaneva A. Concomitant contact allergy to formaldehyde and methacrylic monomers in students of dental medicine and dental patients. Int J Occup Med Environ Health. 2014;27:797–807. 10.2478/s13382-014-0314-4. 25323987 10.2478/s13382-014-0314-4

[28] Maserejian NN, Shrader P, Brown OA, Trachtenberg FL, Soncini J, Hauser R, et al. Dental sealants and composite restorations and longitudinal changes in immune function markers in children. Int J Paediatr Dent. 2014;24:215–25. 10.1111/ipd.12064. 24033362 10.1111/ipd.12064 PMC4593059

[29] Yıldız M, Alp HH, Gül P, Bakan N, Özcan M. Lipid peroxidation and DNA oxidation caused by dental filling materials. J Dent Sci. 2017;12:233–40. 10.1016/j.jds.2017.02.002. 30895056 10.1016/j.jds.2017.02.002 PMC6400006

[30] Berge TLL, Lygre GB, Lie SA, Lindh CH, Björkman L. Bisphenol A in human saliva and urine before and after treatment with dental polymer-based restorative materials. Eur J Oral Sci. 2019;127:435–44. 10.1111/eos.12647. 31392814 10.1111/eos.12647 PMC6790658

[31] Trachtenberg FL, Shrader P, Barregard L, Maserejian NN. Dental composite materials and renal function in children. Br Dent J. 2014. 10.1038/sj.bdj.2014.36. 24457893 10.1038/sj.bdj.2014.36

[32] Maserejian NN, Trachtenberg FL, Hauser R, McKinlay S, Shrader P, Bellinger DC. Dental composite restorations and neuropsychological development in children: Treatment level analysis from a randomized clinical trial. Neurotoxicology. 2012;33:1291–7. 10.1016/j.neuro.2012.08.001. 22906860 10.1016/j.neuro.2012.08.001 PMC3470777

[33] Bakopoulou A, Mourelatos D, Tsiftsoglou AS, Mioglou E, Garefis P. Sister-chromatid exchange, chromosomal aberrations and delays in cell-cycle kinetics in human lymphocytes induced by dental composite resin eluates. Mutat Res Genet Toxicol Environ Mutagen. 2008;649:79–90. 10.1016/j.mrgentox.2007.08.009. 10.1016/j.mrgentox.2007.08.009 17950025

[34] Lyapina M, Dencheva M, Nikolov G, Deliverska M. Biomonitoring of urinary levels of bisphenol A. Compt rendus de l’Acad bulgare des Sci. 2016;69(6):807–14.

[35] Maserejian NN, Hauser R, Tavares M, Trachtenberg FL, Shrader P, McKinlay S. Dental composites and amalgam and physical development in children. J Dent Res. 2012;91:1019–25. 10.1177/0022034512458691. 22972857 10.1177/0022034512458691 PMC3525131

[36] Polydorou O, König A, Hellwig E, Kümmerer K. Long-term release of monomers from modern dental-composite materials. Eur J Oral Sci. 2009;117:68–75. 10.1111/j.1600-0722.2008.00594.x. 19196321 10.1111/j.1600-0722.2008.00594.x

[37] Seiss M, Langer C, Hickel R, Reichl F-X. Quantitative determination of TEGDMA, BHT, and DMABEE in eluates from polymerized resin-based dental restorative materials by use of GC/MS. Arch Toxicol. 2009;83:1109–15. 10.1007/s00204-009-0470-7. 19771414 10.1007/s00204-009-0470-7

[38] Sideridou I, Tserki V, Papanastasiou G. Effect of chemical structure on degree of conversion in light-cured dimethacrylate-based dental resins. Biomaterials. 2002;23:1819. 11950052 10.1016/s0142-9612(01)00308-8

[39] Geurtsen W, Lehmann F, Spahl W, Leyhausen G. Cytotoxicity of 35 dental resin composite monomers/additives in permanent 3T3 and three human primary fibroblast cultures. J Biomed Mater Res. 1998;41:474–80. 10.1002/(SICI)1097-4636(19980905)41:3%3c474::AID-JBM18%3e3.0.CO;2-I. 9659618 10.1002/(sici)1097-4636(19980905)41:3<474::aid-jbm18>3.0.co;2-i

[40] Polydorou O, König A, Hellwig E, Kümmerer K. Long-term release of monomers from modern dental-composite materials. Eur J Oral Sci. 2009;117:68–75. 10.1111/j.1600-0722.2008.00594.x. 19196321 10.1111/j.1600-0722.2008.00594.x

[41] Issa Y, Watts DC, Brunton PA, Waters CM, Duxbury AJ. Resin composite monomers alter MTT and LDH activity of human gingival fibroblasts in vitro. Dent Mater. 2004;20:12–20. 10.1016/S0109-5641(03)00053-8. 14698769 10.1016/s0109-5641(03)00053-8

[42] Van Landuyt KL, Nawrot T, Geebelen B, De Munck J, Snauwaert J, Yoshihara K, et al. How much do resin-based dental materials release? A meta-analytical approach. Dental Mater. 2011;27:723–47. 10.1016/j.dental.2011.05.001. 10.1016/j.dental.2011.05.001 21664675

[43] Wataha JC, Lockwood PE, Bouillaguet S, Noda M. In vitro biological response to core and ¯owable dental restorative materials. n.d. 10.1016/s0109-5641(02)00012-x 12498893

[44] Miguez PA, Pereira PNR, Foxton RM, Walter R, Nunes MF, Swift EJ. Effects of flowable resin on bond strength and gap formation in class I restorations. Dent Mater. 2004;20:839–45. 10.1016/j.dental.2003.10.015. 15451239 10.1016/j.dental.2003.10.015

[45] AlShaafi MM. Factors affecting polymerization of resin-based composites: a literature review. Saudi Dental Journal. 2017;29:48–58. 10.1016/j.sdentj.2017.01.002. 28490843 10.1016/j.sdentj.2017.01.002 PMC5411902

[46] Ferracane JL, Aday P, Matsumoto H, Marker VA. Relationship between shade and depth of cure for light-activated dental composite resins. Dent Mater. 1986;2:80–4. 3458636 10.1016/s0109-5641(86)80057-4

[47] DanilGuiraldo R, Consani S, Leonardo R, Consani X, Bittencourt Berger S, Mendes WB, et al. Light energy transmission through composite influenced by material shades. Bull Tokyo Dent Coll. 2009;50:183. 20179393 10.2209/tdcpublication.50.183

[48] Zorzin J, Maier E, Harre S, Fey T, Belli R, Lohbauer U, et al. Bulk-fill resin composites: Polymerization properties and extended light curing. Dent Mater. 2015;31:293–301. 10.1016/j.dental.2014.12.010. 25582061 10.1016/j.dental.2014.12.010

[49] Garcia D, Yaman P, Dennison J, Neiva GF. Polymerization shrinkage and depth of cure of bulk fill flowable composite resins. Oper Dent. 2014;39:441–8. 10.2341/12-484-L. 24304339 10.2341/12-484-L

[50] Harlow JE, Rueggeberg FA, Labrie D, Sullivan B, Price RB. Transmission of violet and blue light through conventional (layered) and bulk cured resin-based composites. J Dent. 2016;53:44–50. 10.1016/j.jdent.2016.06.007. 27373167 10.1016/j.jdent.2016.06.007

[51] Moszner N, Fischer UK, Ganster B, Liska R, Rheinberger V. Benzoyl germanium derivatives as novel visible light photoinitiators for dental materials. Dent Mater. 2008;24:901–7. 10.1016/j.dental.2007.11.004. 18155290 10.1016/j.dental.2007.11.004

[52] Bucuta S, Ilie N. Light transmittance and micro-mechanical properties of bulk fill vs. conventional resin based composites. Clin Oral Investig. 2014;18:1991–2000. 10.1007/s00784-013-1177-y. 24414570 10.1007/s00784-013-1177-y

[53] Kingman A, Hyman J, Masten SA, Jayaram B, Smith C, Eichmiller F, et al. Bisphenol A and other compounds in human saliva and urine associated with the placement of composite restorations. J the Am Dental Assoc. 2012;143:1292–302. 10.14219/jada.archive.2012.0090. 10.14219/jada.archive.2012.0090 PMC12266333 23204083

[54] Goldberg M. In vitro and in vivo studies on the toxicity of dental resin components: a review. Clin Oral Investig. 2008;12:1–8. 10.1007/s00784-007-0162-8. 18040729 10.1007/s00784-007-0162-8

